# 1939. Who to Boost When: An Analysis of Dosing Interval and Age on COVID-19 Outcomes in the COVE Trial During the Delta and Omicron Waves

**DOI:** 10.1093/ofid/ofad500.2470

**Published:** 2023-11-27

**Authors:** Dean Follmann, Xiaowei Wang, Peter Gilbert, Lindsey R Baden, Hana M El Sahly, Brandon Essink, Mary Marovich, Holly Janes, Weiping Deng, Frances Priddy, Avika Dixit, Joanne Tomassini, Rituparna Das, Jacqueline Miller, Honghong Zhou

**Affiliations:** National Institute of Allergy and Infectious Disease, Bethesda, MD; Moderna, Inc., Cambridge, Massachusetts; Fred Hutchinson Cancer Center, Seattle, Washington; Brigham and Women's Hospital, Boston, MA; Baylor College of Medicine, Houston, TX; Meridian Clinical Research, Omaha, Nebraska; National Institute of Allergy and Infectious Diseases, National Institutes of Health, Bethesda, Maryland; Fred Hutchinson Cancer Research Center, Seattle, WA; Moderna, Inc., Cambridge, Massachusetts; Moderna, Inc., Cambridge, Massachusetts; Moderna, Inc., Cambridge, Massachusetts; Moderna, Inc., Cambridge, Massachusetts; Moderna, Inc., Cambridge, Massachusetts; Moderna, Inc., Cambridge, Massachusetts; Moderna, Inc., Cambridge, Massachusetts

## Abstract

**Background:**

The effects of age and interval between vaccine doses on COVID-19 are incompletely understood, yet critical for public health policy.

**Methods:**

This analysis evaluated the effectiveness of an mRNA-1273 50 μg booster (3^rd^ dose) in ∼17,000 boosted participants in the phase 3 coronavirus efficacy (COVE) trial who previously received 2-doses of 100 µg mRNA-1273 during the randomized placebo-controlled phase (mRNA-1273, 1st dose July-Oct 2020), or open label phase (placebo-mRNA-1273, 1st dose Dec 2020-Apr 2021). A single 50 µg mRNA-1273 (original vaccine) booster was administered starting Sep 2021. The effect of boosting on COVID-19 risk was assessed in Cox models by the initially randomized arm, time since booster, and age groups (< 65, ≥ 65) between Sep 2021-May 2022 during the open label phase of COVE.

**Results:**

The median dosing intervals (IQR) between primary series and booster were 8.2 (7.8-8.7) months for the placebo-mRNA-1273 and 12.9 (12.3-13.5) months for the mRNA-1273 arms. A booster dose provided substantial additional protection against COVID-19 during the Delta wave through 60 days and was high for Omicron (BA.1), then waned over time by 110 days (Figure). The risk of Omicron COVID-19 post booster was lower by 24% (95% CI: 16%, 32%) in the mRNA-1273 vs the placebo-mRNA arm indicating possible benefit of longer boost interval. In a separate analysis, among participants ≥ 65 years, the initial booster (95% CI) efficacy against Omicron was 86% (69%, 93%) which waned to 28% (-47%, 65%) after ∼4 months. For those < 65 years, the analogous estimates were 50% (36%, 61%) and 6% (-29%, 31%), indicating a larger boosting effect in those ≥ 65 years. When further stratified by arm, for those ≥ 65 years, the initial boost efficacy against Omicron was 79% (41%, 92%) for the placebo-mRNA arm and 91% (79%, 96%) for mRNA-1273. For those < 65, the analogous estimates were 52% (30%, 62%) and 55% (39%, 66%), suggesting the effect of booster interval was largely concentrated in those ≥ 65 years.

**Figure:**
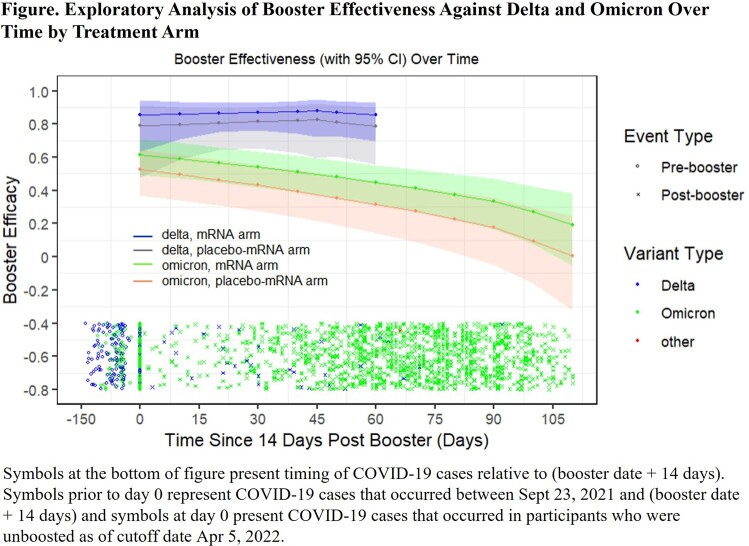

**Conclusion:**

Boosting with mRNA-1273 provided additional COVID-19 protection during the Delta and Omicron variant waves. Overall, boosting reduced the risk of Omicron COVID-19 but waned over time (∼4 months). The effects of boosting and interval were more pronounced in those ≥ 65 years of age.

**Disclosures:**

**Xiaowei Wang, PhD**, Moderna, Inc.: Stocks/Bonds **Peter Gilbert, PhD**, NIH NIAID: Grant/Research Support **Lindsey R. Baden, MD**, NIH/NIAID: Grant/Research Support **Hana M. El Sahly, MD**, NIH and/or NIAID: Grant/Research Support **Holly Janes, PhD**, National Institutes of Health: Grant/Research Support **Weiping Deng, PhD**, Moderna, Inc.: Stocks/Bonds **Frances Priddy, MD, MPH**, Moderna, Inc.: Stocks/Bonds|Moderna, Inc.: Stocks/Bonds **Avika Dixit, MBBS**, Moderna, Inc.: Stocks/Bonds **Joanne Tomassini, Ph.D.**, Moderna, Inc.: Advisor/Consultant **Rituparna Das, M.D.**, Moderna, Inc.: Employee|Moderna, Inc.: Stocks/Bonds **Jacqueline Miller, MD**, Moderna, Inc.: Employee|Moderna, Inc.: Stocks/Bonds **Honghong Zhou, Ph.D.**, Moderna, Inc.: Stocks/Bonds

